# The clinical efficacy of traditional Chinese medicine in the treatment of rheumatoid arthritis with interstitial lung disease: A protocol of systematic review and meta-analysis

**DOI:** 10.1097/MD.0000000000022453

**Published:** 2020-10-09

**Authors:** Zhaoyi Liu, Jie Shen, Zhouli Shen, Dongyi He

**Affiliations:** Department of Rheumatology and Immunology, GuangHua Hospital of Integrated Traditional Chinese and Western, Shanghai, China.

**Keywords:** interstitial lung disease, protocol, rheumatoid arthritis, systematic review, traditional Chinese medicine

## Abstract

**Background:**

The objective of this meta-analysis was to summarize and identify the available evidence from studies to estimate the clinical value of traditional Chinese medicine (TCM) in the treatment of rheumatoid arthritis with interstitial lung disease (RA-ILD). And provides clinicians with evidence on which to base their clinical decision making.

**Methods:**

This review will include all studies comparing clinical efficacy of TCM in the treatment of RA-ILD. The search strategy will be performed in 9 databases. We will not establish any limitations to language and publication status, published from inception to the August 2020. Two reviewers will screen, select studies, extract data, and assess quality independently. Outcome is lung function, number of swelling joints, number of painful joints, duration of morning stiffness, VAS score, adverse effects, quality of life, ESR, CRP, rheumatoid factor and safety. The methodological quality including the risk of bias of the included studies will be evaluated. We will carry out statistical analysis using RevMan 5.3 software.

**Results:**

This study will summarize current evidence to assess the efficacy and safety of TCM in the treatment of RA-ILD.

**Conclusion:**

The findings of this study will provide helpful evidence for the clinician, and will promote further studies, as well as studying the value of TCM.

**Registration number:**

INPLASY202080108 (DOI number: 10.37766/inplasy2020.8.0108).

## 1 Introduction

Rheumatoid arthritis (RA), which affects approximately 1% of the population in developed countries, is a systemic inflammatory disorder that impacts diarthrodial joints, causing progressive, symmetric, and erosive destruction of the cartilage and bone. Although the joints represent a primary target, extra-articular manifestations of disease are frequent, with an estimated prevalence of 40%. Lung involvement is particularly common, with potential effects on serosal, airway, and/or parenchymal tissue. And the interstitial lung disease (ILD) is potentially the most devastating pulmonary issue. However, this complication is underappreciated, particularly in its earliest stages. Therefore, novel treatments are urgently demanded.^[1–6]^

For centuries, herbal medicine has been used as a complementary and alternative medicine for western medicine in China. Although TCM have been used clinically in the treatment of RA for many years, the efficacy and safety still need evidence-based medical research.^[7–10]^ To the best of our knowledge, there is no meta-analysis analysis the clinical efficacy of TCM for RAI- ILD. Consequently, the objective of this meta-analysis was to summarize and identify the available evidence from these studies to estimate the clinical value of TCM. And provides clinicians with evidence on which to base their clinical decision making.

## 2 Methods

### 2.1 Study registry

The protocol was registered on the International Platform of Registered Systematic Review and Meta-analysis Protocols (INPLASY202080108). The preferred reporting items for systematic review and meta-analysis protocols (PRISMA) will serve as guidelines for reporting present review protocol and subsequent formal paper.^[11]^

### 2.2 Eligibility criteria for including studies

#### 2.2.1 Types of studies

We will include all studies comparing the TCM in the treatment of RA-ILD, including observational study and RCT. Any other types of studies, such as animal studies, case reports, case series and review will all be excluded.

#### 2.2.2 Types of interventions

##### 2.2.2.1 Experimental group

All patients in the experimental group received TCM for their treatment in this study (including oral Chinese medicine or external application of Chinese medicine).

##### 2.2.2.2 Control group

The participants in the control group could receive any other treatments in this study

#### 2.2.3 Types of patients

Patients suffered from RA-ILD will be included without sex, age, course, ethnicity, disease duration or disease severity restrictions.

#### 2.2.4 Types of outcome measurements

Primary outcomes.

1. Lung function.
2. The number of swelling joints affected by RA.
3. The number of painful joints affected by RA.
4. The duration of morning stiffness.

Secondary outcomes.

1. Pain visual analog scale (VAS) score.
2. Physician VAS score.
3. Adverse effects.
4. Quality of life.
5. Erythrocyte sedimentation rate.
6. C reactive protein.
7. Rheumatoid factor.
8. Safety.

### 2.3 Literature sources and search

We will perform literature searches using the following electronic bibliographic databases from their inception onwards to the August 2020: MEDLINE, Springer, Web of Science, PubMed, EMBASE, the Cochrane Central Register of Controlled Trials, Evidence Based Medicine Reviews, VIP, and CNKI. We will not establish any limitations to language and publication status. The following electronic databases were searched from their inception dates through August 2020. The search terms were integrated as follows: “∗rheumatoid arthritis∗ AND ∗interstitial lung disease∗ AND (∗Traditional Chinese Medicine∗ OR ∗Traditional Chinese Medicine Formula∗ OR ∗Chinese Herb Formula∗ OR ∗Chinese herbal drug∗)”.

### 2.4 Study selection

**Figure 1:**
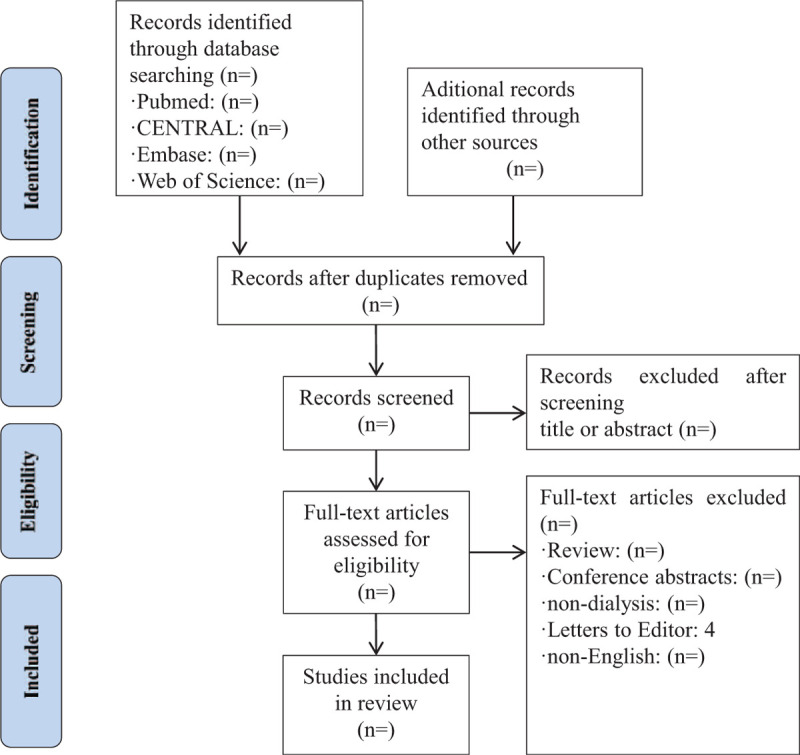
Study Flow.

All duplicated studies will be imported into Endnote X7 software and excluded before the screening. Two authors will independently scan all the records from title and abstract and all irrelevant literatures will be removed. Then, full manuscripts of all remaining studies will be further identified to check if they meet all inclusion criteria. We will note all excluded citations with specific reasons. If there are any different opinions between 2 authors, we will invite another author for consultation and final decision will be made after discussion. The detail of the study selection will be presented in a PRISMA flow diagram (Fig. 1)

#### 2.4.1 Data extraction

Two authors will independently extract the following associated information from each included trial: first author, time of publication, sample size, randomization methods, blinding, concealment, allocation, details of intervention and controls, duration of follow-up, outcome measurement tools, and any other relevant information. A third senior author will help to reconcile any divergences between 2 authors.

#### 2.4.2 Missing data dealing with

If we identify any unclear or missing data, we will contact original authors to obtain them. If we cannot get reply, we will only analyze available data and will discuss its potential affect as limitation.

#### 2.4.3 Quality assessment

The Cochrane risk of bias tool, which is recommended by the Cochrane Reviewers Handbook 5.0.24, will be used to evaluate the quality of the included studies. Two independent reviewers will evaluate the quality of selected articles from the following 5 aspects: selection bias (random sequence generation or allocation concealment), performance bias and detection bias (blinding), attrition bias (incomplete outcome data), reporting bias (selective outcome reporting), and other biases. If necessary, we will contact the corresponding author to clarify issues. The result of the consistency evaluation will be presented with Kappa statistics, Kappa value <0.75 will be considered the consistency have reached. Any disagreements will be resolved through discussion or consultation.

#### 2.4.4 Subgroup analysis

We will preside over subgroup analysis to explore any potential heterogeneity and inconsistency based on the different factors.

#### 2.4.5 Sensitivity analysis

We will consider running sensitivity analysis to identify the robustness and stability of merged results by excluding studies with high risk of bias.

#### 2.4.6 Reporting bias

If necessary, we will examine the reporting bias using funnel plot and Egger regression test when >10 trials are included.

### 2.5 Data synthesis

We will undertake RevMan 5.3 software to analyze data and to perform meta-analysis if it is necessary. We will calculate all continuous data using mean difference or standardized mean difference and 95% confidence intervals. As for dichotomous data, we will exert it using risk ratio and 95% CI. The heterogeneity as determined by the Cochran statistics was <0.10 of the *χ*^2^ test. If the *I*^2^ value was >50%, we marked it as a considerable level of heterogeneity; otherwise, we considered it to be a good homogeneity. We also assessed clinical heterogeneity. Statistically and clinically homogeneous studies were pooled using a fixed-effects model; otherwise, a random-effects model was used when the heterogeneity was significant. Additionally, subgroup analysis will be operated to explore any possible reasons for the high heterogeneity. Whenever it is possible, we will conduct meta-analysis if at least 3 eligible criteria are fulfilled. Otherwise, meta-analysis will not be carried out if only 1 or 2 studies meet the inclusion criteria. Under such situation, the findings will be presented in a narrative summary. We will perform narrative synthesis if running meta-analysis is inappropriate due to the high heterogeneity. All narrative descriptions will be carried out based on the Guidance on the Conduct of Narrative Synthesis in Systematic Reviews.

## 3 Discussion

Several predictors of mortality have been identified in RA-ILD. Age is the most consistent variable that has been identified as a significant predictor of poor prognosis across multiple studies. More recently, women with seropositive RA have been shown to have a nearly 3-fold increased risk of mortality due to respiratory disease (including chronic obstructive pulmonary disease, asthma, pleurisy, lung abscess, bronchiectasis, and pulmonary fibrosis) compared to women without RA.^[12–14]^

The optimal treatment for RA-ILD has not been determined and is largely based on data derived from other CTD-ILDs, primarily SSc-associated ILD. Therefore, careful attention should be made to the baseline assessment of disease severity, presentation (acute, subacute, and chronic), and risks and benefits of therapy for each individual. In general, treatment should be considered in patients with clinical, functional, or radiologic deterioration, and histopathologic patterns other than non-usual interstitial pneumonia.^[15–18]^

The strength of this systematic review and meta-analysis will include: search a comprehensive range of databases, including Chinese and English databases, more rigorous and detailed concerning quality assessment and data extraction. In addition, the findings obtained in the present study will provide helpful evidence in clinical practice. Furthermore, it will also help to promote further studies and clarify the direction for the future research.

On the contrary, this study has several potential limitation. There may be a language bias, although there is not language limitation in this study. Moreover, there may be a large heterogeneity, which may bias the results.

## Author contributions

ZYL JS, and ZLS conducted of the protocol and drafting the manuscript. All authors participated in the design of the study. DYH is co-corresponding author of this manuscript. All authors read and approved the final manuscript.

**Conceptualization:** Zhaoyi Liu.

**Data curation:** Zhaoyi Liu, Jie Shen.

**Formal analysis:** Dongyi He.

**Funding acquisition:** Zhouli Shen, Dongyi He.

**Investigation:** Zhouli Shen.

## Abbreviations

Abbreviations: TCM = traditional Chinese medicine, RA = rheumatoid arthritis; ILD= interstitial lung disease.
